# Polyelectrolyte-Assisted Dispersions of Reduced Graphite Oxide Nanoplates in Water and Their Gas-Barrier Application

**DOI:** 10.1021/acsami.1c08889

**Published:** 2021-09-03

**Authors:** Lorenza Maddalena, Tobias Benselfelt, Julio Gomez, Mahiar Max Hamedi, Alberto Fina, Lars Wågberg, Federico Carosio

**Affiliations:** † Dipartimento di Scienza Applicata e Tecnologia, Politecnico di Torino, Alessandria Campus, Viale Teresa Michel 5, 15121 Alessandria, Italy; ‡ Department of Fibre and Polymer Technology, KTH Royal Institute of Technology, Teknikringen 58, SE-100 44 Stockholm, Sweden; § AVANZARE Innovacion Tecnologica S.L., 26370 Navarrete, La Rioja, Spain

**Keywords:** water-dispersion of graphene, polyelectrolyte, rGO, gas barrier, layer-by-layer, single stagnation point adsorption reflectometry

## Abstract

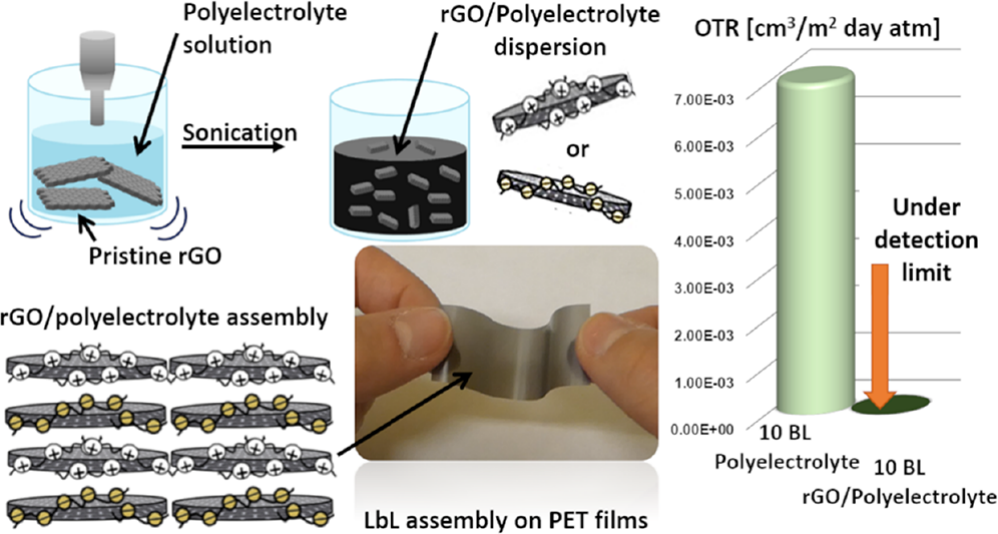

Dispersion of graphene and related materials in water is needed to enable sustainable processing of these 2D materials. In this work, we demonstrate the capability of branched polyethylenimine (BPEI) and polyacrylic acid (PAA) to stabilize reduced graphite oxide (rGO) dispersions in water. Atomic force microscopy colloidal probe measurements were carried out to investigate the interaction mechanisms between rGO and the polyelectrolytes (PEs). Our results show that for positive PEs, the interaction appears electrostatic, originating from the weak negative charge of graphene in water. For negative PEs, however, van der Waals forces may result in the formation of a PE shell on rGO. The PE-stabilized rGO dispersions were then used for the preparation of coatings to enhance gas barrier properties of polyethylene terephthalate films using the layer-by-layer self-assembly. Ten bilayers of rGO_BPEI_/rGO_PAA_ resulted in coatings with excellent barrier properties as demonstrated by oxygen transmission rates below detection limits [<0.005 cm^3^/(m^2^ day atm)]. The observed excellent performance is ascribed to both the high density of the deposited coating and its efficient stratification. These results can enable the design of highly efficient gas barrier solutions for demanding applications, including oxygen-sensitive pharmaceutical products or flexible electronic devices.

## Introduction

In the last 20 years, graphene and graphene-related materials (GRMs) have attracted a lot of attention from both the scientific and industrial communities, due to their fascinating properties such as excellent electrical and thermal conductivity,^1^ coupled with outstanding mechanical properties.^2^ GRMs can be classified based on the number of stacked layers, the lateral size, and the carbon-to-oxygen content ratio.^3^ While graphene is defined as a monolayer of sp^2^ carbon atoms arranged in a honeycomb structure, the most commonly applied GRMs are graphene oxide (GO) (partially oxidized monolayer), few-layer graphene (FLG, 2–10 layers), graphite nanoplates (GNPs, >10 layers), and graphite oxide (GO) nanoplates (partially oxidized GNPs).^4^ Oxidized GRMs are often used as intermediates in material preparation, exploiting their affinity to water or polar solvents, and can be subsequently reduced via chemical or thermal treatments, to partially restore the mechanical, thermal, and electrical properties of pristine GRMs. GRMs are prepared by a wide range of techniques.^1,5^ Among the top-down approaches, the liquid phase exfoliation (LPE) of graphite is one the most investigated and useful techniques for the production of graphene, FLG, and GNP in large amounts.^6−8^ Typical solvents that are known to allow stable dispersions of graphene and its multilayers are *N*-methyl-2-pyrrolidone (NMP), *N*-cyclo-2-pyrrolidone, dimethylformamide, and dimethyl sulfoxide.^7^ However, the use of such organic solvents brings several drawbacks; NMP can degrade and polymerize during the procedure, which changes the viscosity of the dispersion and thus limits its exfoliating ability.^9,10^ The high boiling point of organic solvents furthermore makes their removal difficult. Another concern is the known or suspected toxicity of some of these solvents^11^ and their subsequent purification and reuse. In contrast, water is considered a safe and highly desirable alternative for dispersing graphene. Unfortunately, pure water cannot disperse graphene and requires the introduction of additives or stabilizers (e.g., sodium cholate, pyrene, or perylene derivatives) to compensate for the difference in surface energy between water and graphene-like surfaces.^12−18^ While surfactants may allow water-based processing, their removal in postprocessing steps at the intended interface is, however, difficult and may affect the properties of obtained materials and devices. Tip sonication in an aqueous solution of polyelectrolytes (PEs) also produces reduced GO (rGO) nanoplate dispersions stabilized by PE (rGO_PE_) with a processing capability typical of PE solutions, including layer-by-layer (LbL) assembly, as also described in the present work. The most employed approach is to prepare PE dispersions of rGO nanoplates, where PEs, such as poly(sodium 4-styrenesulfonate)^19^ or poly(diallyldimethylammonium chloride),^20^ are added to a rGO–LPE dispersion during the reduction treatment. Conversely, in this work, we investigate a procedure where the rGO dispersion in water is mediated by PEs. The obtained stable dispersions are further employed to fabricate functional coatings using LbL self-assembly. The LbL method is based on the alternate adsorption of anionic and cationic colloids, or polymers/PEs, onto a substrate, mainly using an ion-exchange process driven by the entropy of released counterions.^21,22^ The process is affected by several parameters such as the nature of the employed PE,^21^ the temperature,^23^ the pH,^24^ the ionic strength,^25^ and the nature of the counterions.^22,26^ LbL assembly has been used to fabricate numerous graphene-based multilayer nanocomposites^27^ with thicknesses in the range of 10–1000 nm,^23,24,28^ and different functional groups have been added to graphene to influence the surface chemistry of the nanocomposites and change, for example, its wettability^29^ or gas barrier properties.^30−36^ Moreover, when nanoplates such as clays, layered double hydroxides,^37−39^ or GRM are employed, the obtained nanostructured coatings exhibit a “brick-and-mortar” organization, where nanoparticles (bricks) are embedded in a polymer matrix (mortar) with a preferential orientation parallel to the substrate surface.^40,41^ Up to now, mostly GO nanoplatelet suspensions have been employed to build multilayer LbL coatings.^42^ For example, Yu et al. deposited 5 BLs comprising branched polyethylenimine (BPEI) and GO obtaining oxygen transmission rates (OTRs) lower than 0.005 cc/m^2^ day atm.^42^ Further studies proved that the pH and concentration of GO could influence the achieved barrier properties while also enabling the use of GO containing LbL coatings as selective membranes.^33,43^ The hydrophilic nature of GO cannot, however, prevent swelling in humid environments, thus resulting in detrimental gas barrier performances at a high relative humidity (RH).^44^ GO reduction under mild conditions (175 °C for 90 min) has been proposed as a possible solution, allowing maintaining the achieved performances even under 100% RH conditions.^32^ A drawback of this approach is related to the limited transparency of the film after reduction. Recently, cetyltrimethylammonium bromide-stabilized rGO was LbL assembled with polyvinyl alcohol resulting in a high-performance gas barrier membrane (−92% OTR reduction compared to polyethylene terephthalate (PET)) with exceptional optical transparency.^45^ Although not as efficient as the previously mentioned GO containing LbL assemblies, the gas barrier potential of rGO had been clearly shown in the mentioned work. To fully disclose the potentialities of rGO, we used water solutions of polyacrylic acid (PAA) and BPEI to stabilize rGO nanoplates in water (thus obtaining rGO_PE_ dispersions) by a mild sonication process to enable the LbL assembly. A previous pioneering work performed by Lu et al. demonstrated the potentialities of PE in stabilizing GNPs.^46^ Of the selected PEs, only BPEI was found to be capable of yielding stable (24 h) suspensions suitable for LbL assembly. However, the mechanism behind the stabilizing effects of PEs is not yet well understood.^46^ Conversely, here we report a viable and easy strategy for the preparation of stable (up to 12 months) rGO_PE_ dispersions. Moreover, to obtain fundamental knowledge on the stabilization mechanism, an atomic force microscopy (AFM) colloidal probe was used to investigate the interaction between the PEs and a graphene model surface. The possibility of employing the obtained rGO_PE_ dispersions for the preparation of LbL-assembled nanocomposite coatings was also investigated. The coating growth was monitored by infrared spectroscopy, quartz crystal microbalance with dissipation (QCM-D), stagnation point adsorption reflectometry (SPAR), and field-emission scanning electron microscopy (FESEM). Compared with the coatings made of only PEs, the presence of rGO nanoplates leads to thinner assemblies, in which rGO nanoplates are highly oriented parallel to the surface of the substrate. This novel LbL approach, where PE-stabilized nanoplates are deposited in every deposition step, was applied to prepare gas barrier coatings onto 10 μm thick PET films, demonstrating significant reductions in oxygen permeability at very limited rGO nanoplate concentrations due to the high degree of orientation of the platelets.

## Results and Discussion

### PE-Stabilized rGO Dispersions

**Figure 1:**
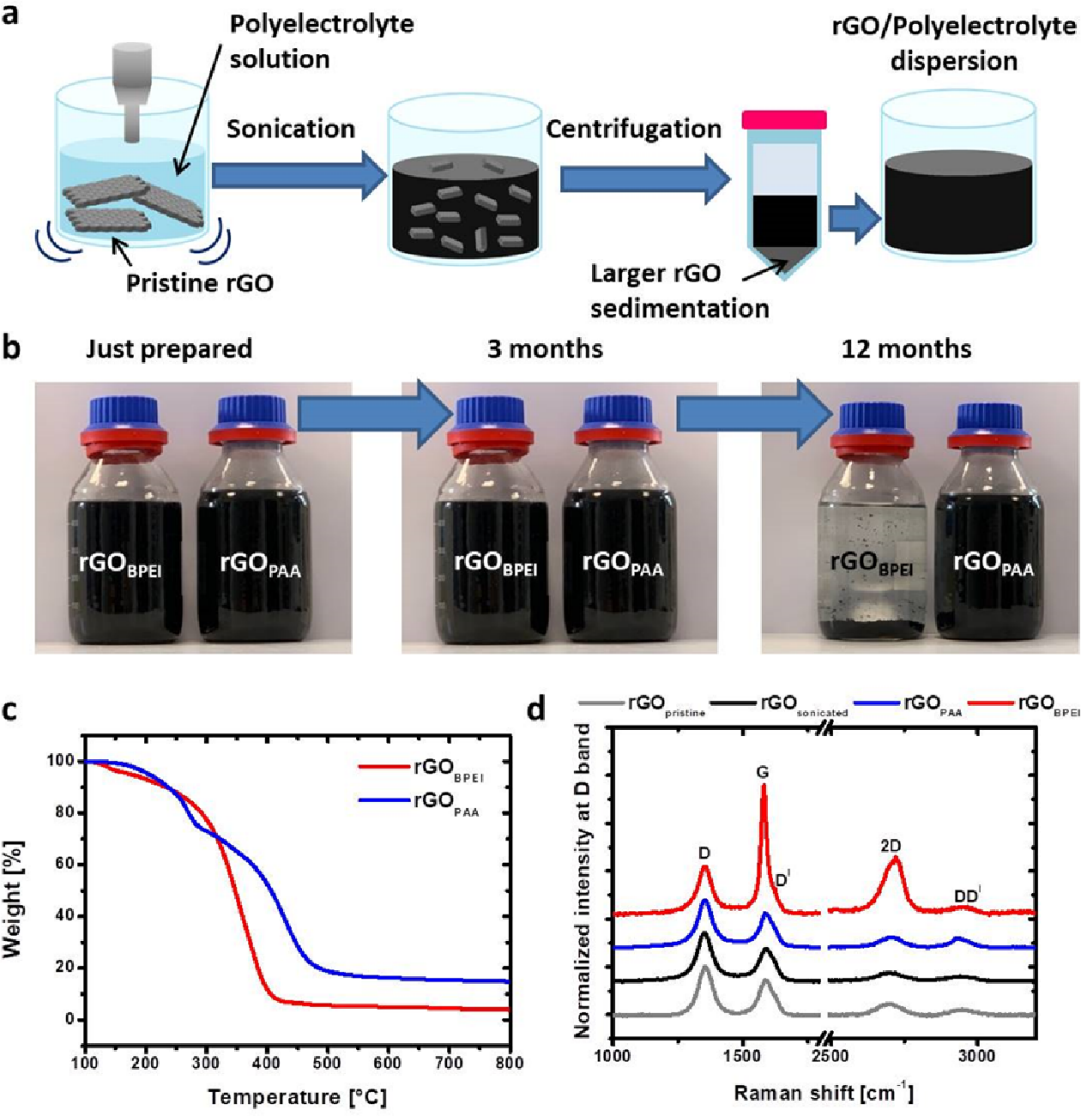
Schematic of rGO-PE dispersion preparation (a). Pictures of rGO_BPEI_ and rGO_PAA_ dispersions at different times (b). TGA measurements of dried rGO_BPEI_ and rGO_PAA_ in a N_2_ atmosphere (c) and Raman spectra of powder pristine rGO, dried dispersions of rGO, rGO_BPEI_, and rGO_PAA_ (d).

Colloidal dispersions of BPEI-stabilized rGO (rGO_BPEI_) and PAA-stabilized rGO (rGO_PAA_) were prepared and characterized to determine the concentration, dimensions, and the interaction of the platelets with the PE in water. Figure 1a schematizes the applied procedure for dispersing rGO in water. Figure 1b shows the photographs of the prepared dispersion after up to 12 months of storage under static conditions.

Both rGO_BPEI_ and rGO_PAA_ dispersions had very high stability over time at room temperature, with no significant precipitation after several weeks. Differences in stability between the two dispersions are observable after 3 months. During long storage time, rGO_BPEI_ progressively sediments, leading to almost complete precipitation after 12 months, whereas rGO_PAA_ appears to be fully stable even after 1 year of storage. The rGO nanoplate concentration was evaluated by TGA, following the procedure described in the Characterization section (Figure 1c). The stability of neat components was first evaluated showing that rGO nanoplates have a negligible weight loss in the considered range of temperatures, while PAA and BPEI decompose leaving final residues of 13 and <1 wt %, respectively (Figure S2 and Table S1). The rGO concentration in the colloidal dispersions was estimated to be 0.002 and 0.004 wt % for rGO_PAA_ and rGO_BPEI_, respectively (initial value, 0.025 wt %). From the obtained data, it is clear that the ratio between the rGO and the PE in the final dispersions is very low, reflecting the fraction of larger rGO particles removed during centrifugation.

Raman spectroscopy provides additional insight into the quality, amount, distance, and nature of structural defects of the dispersed rGO nanoplates, including edge defects, grain boundaries, vacancies, heteroatoms, and sp^3^ carbon (Figure 1d). The Raman spectra obtained from the dried rGO (as received powder) dispersion, obtained under the same conditions as those of rGO_BPEI_ and rGO_PAA_ dispersions (labeled as rGO), show two sets of signals: the first includes the D, G, and D^I^ bands, while the second is composed of the 2D and DD^I^ bands. In the first set, the D band corresponds to the breathing mode of six-atom rings and requires defects for its activation, while the G band corresponds to the in-plane stretching vibration mode of sp^2^ carbon atoms.^47^ In the second set, 2D and DD^I^ are the overtone signals of D and D + D^I^ bands, respectively. The presence of broad D and G bands is associated with the presence of defect signals and is the result of the overlap of different interbands, such as D″, D, D*, G, and D^I^.^48^ A decrease in the defect concentration in the graphene materials is associated with a decrease in the FWHM of D and G bands in rGO.^49,50^ This spectrum is very similar to that observed for the pristine rGO powder (gray curve in Figure 1d) except for the *I*(D)/*I*(G) ratio that slightly increases from 1.30 ± 0.07 to 1.45 ± 0.02 and a small reduction of FWHM of D and G bands, which can be explained by an increase in grain boundary defects as a consequence of the tip sonication.^5,51^ The same considerations are valid for rGO_PAA_ Raman spectra, where *I*(D)/*I*(G) is mostly unaltered with respect to rGO, suggesting that the presence of PAA does not affect the defectiveness of suspended rGO in PAA.^52^ However, for rGO_BPEI_, the G band shifted to lower wavenumbers compared to rGO. In addition, the decrease in *I*(D)/*I*(G) to 0.38 ± 0.03, the decrease in DD^I^ and *I*(DD′)/*I*(2D), and the shift to the higher wavenumber of the 2D′ band^51^ could be explained by an increase in the average distance between defects, *L*_D_, calculated by using the Cançado relationship.^50^*L*_D_ increased from 10 nm in rGO to 20 nm in rGO_BPEI_ due to a reduction process in the presence of BPEI,^5,51^ in agreement with the previously described *in situ* reduction in GO by polyethylenimine.^53^ However, in our case, a more pronounced reduction is observed. In addition, the significant decrease in the FWHM of the G band to a sharp peak is also indicative of a transition from stage 2 (crystalline structures comprising nanocrystalline graphite to low sp^3^ amorphous carbon) of defects in GO to stage 1 (crystalline structures comprising graphite to nanocrystalline graphite) of defects.^53^

Scanning electron microscopy (SEM) and AFM were used to further characterize the dimensions of the dispersed rGO, as shown in Figure S2. SEM measurements performed with dried rGO_BPEI_ and rGO_PAA_ dispersions showed wrinkled rGO flakes with an average length of 3 μm (Figure S3a,b), resulting from the fragmentation of larger flakes observable in pristine rGO powder (Figure S1). The thickness of neat and PE-coated rGO was determined by tapping-mode AFM. The neat rGO simply sonicated in water under the same conditions as those used for rGO_BPEI_ and rGO_PAA_ dispersions leads to closely packed sheets with a thickness between 8 and 10 nm (Figure S3c). Differently from rGO, in the case of rGO_BPEI_ and rGO_PAA_, the surface of the silicon wafer is mostly covered by the rGO embedded in PEs, which is consistent with a rougher surface, preventing the precise estimation of the rGO thickness in both dispersions (Figure S3d,e).

### Fundamentals of the PE–Graphene Interaction

**Figure 2:**
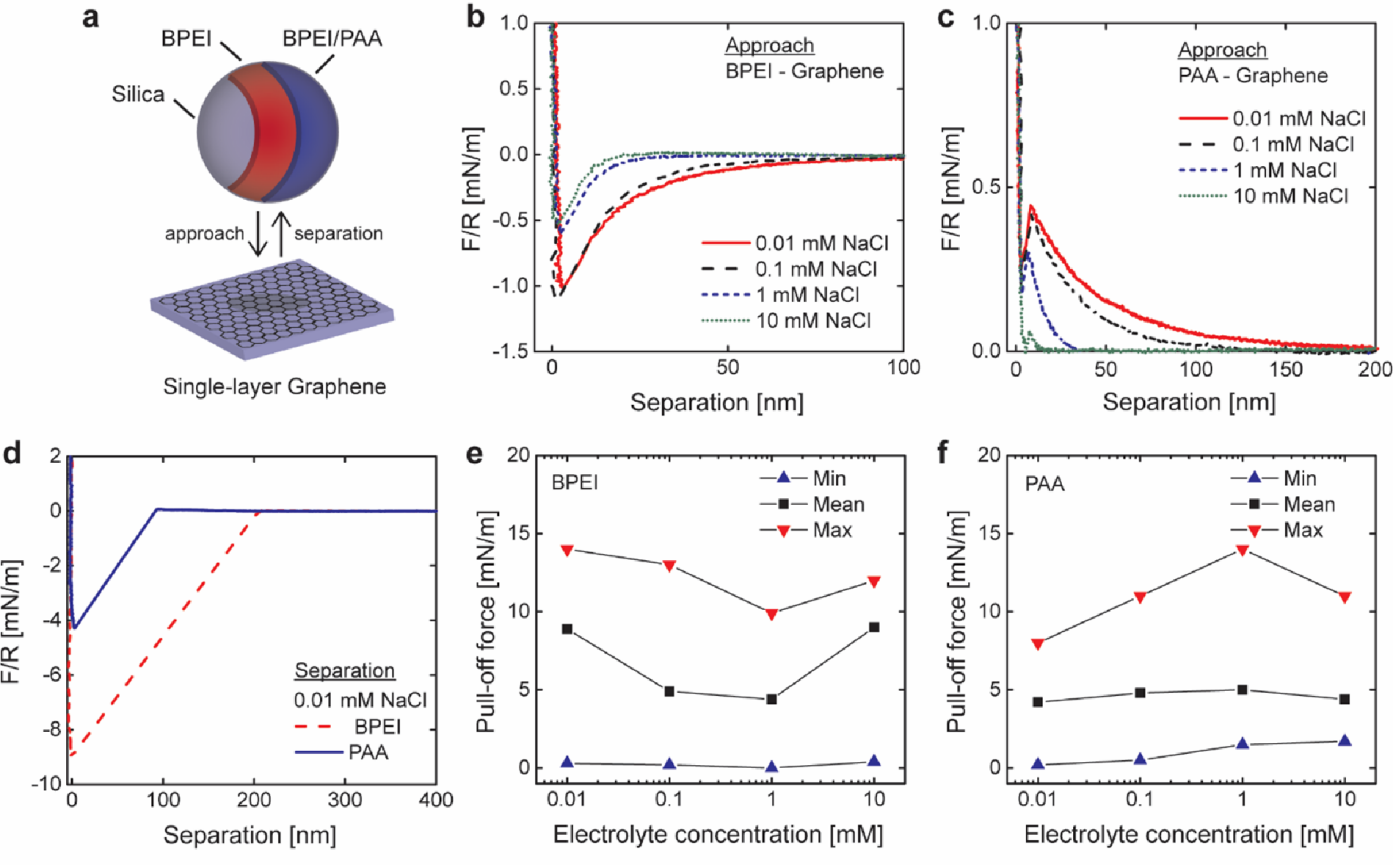
AFM colloidal probe measurements used to study the interaction between graphene and BPEI or PAA. Illustration of the experimental setup (a). Force on approach (*F*), normalized to the radius of the particle (*R*), between BPEI and graphene (b) and PAA and graphene (c). Typical force curves on separation representing adhesion (d). Pull-off force as a function of electrolyte concentration in the case of BPEI (e) and PAA (f). Vertical line at zero separation represents the hard wall contact where the force is proportional to the spring constant of the cantilever according to Hook’s law. The straight line from pull-off to baseline in (d) represents a snap-off (jump from adhesion to zero force).

To study the interactions between the rGO and the PEs, we used AFM colloidal probe measurements. A single-layer graphene on a silica wafer was used as a model surface, and activated silica (Si–O^–^) particles with a radius of 5 μm were attached to a tipless cantilever and coated with BPEI or a BL of BPEI/PAA to be used as probes representing the PEs (Figure 2a). Force curves were recorded in water for both approach and separation under different electrolyte concentrations. Changing the electrolyte concentration provides insights into the nature of the interaction, that is, whether it is dominated by “electrostatic” interactions, van der Waals interactions, or something else.

As a reference, the force between an uncoated silica probe and the graphene surface was recorded (Figure S4). The force curves show repulsion (positive force) on both approach and separation due to the negative charge of the activated silica particle that leads to double-layer repulsion. No adhesion was observed, which is probably due to hydration forces, from the bound water on the highly hydrated silica surface that significantly reduced van der Waals interactions.^54^

In the case of a PE-coated probe, the situation is different, and Figure 2b,c shows attraction on approach in the case of BPEI (negative force) and repulsion on approach in the case of PAA (positive force). The reported plots, the extensive range of the interaction (100–200 nm), and the distinct influence of the electrolyte concentration (less interaction at higher salt concentration) lead to the conclusion that the interaction on approach is electrostatic and is governed by the double layers of the probe and the surface. Figure 2c also shows that in the case of PAA, there is a jump into contact at around 10–20 nm, which represents the force required to overcome the double-layer repulsion and reach a separation distance where van der Waals forces dominate. Note that in this case, hydration forces are small or not present.

The findings in Figure 2b,c show that the graphene surface obtains a negative surface potential when placed in an electrolyte solution, which has been previously observed.^55^ The phenomenon that uncharged interfaces in water obtain a negative potential is common in colloidal science,^56^ but the reason for this is not completely understood. The most accepted explanation is that anions are more polarizable than cations due to their excess electrons and are thus more prone to adsorb onto interfaces.^57^ Another related property is the perturbation of water, which is often discussed in terms of Hofmeister series or specific ion effects, in which large ions with a low charge density (often referred to as chaotropic ions) break the hydrogen-bonded network of water and therefore more favorably reside at the interface between water and another medium.^58,59^ The outcome is that anions, such as OH^–^ or Cl^–^, adsorb onto graphene, and this results in a negative surface charge in water. The issue is that this is a metastable state since dissolved gas even more preferably nucleates at the graphene–water interface in the form of nanobubbles, which over time leads to the long-range capillary attraction commonly observed between hydrophobic surfaces in water.^55,60,61^

A more recent explanation for the charging of uncharged surfaces in water is contact electrification, commonly known as static electricity. In the same way as electrons can be transferred between solid objects when a force is applied, for example, when rubbing a balloon against hair, so can electrons be transferred between a liquid and a solid object.^62,63^ It was recently shown that ion and electron transfers occur simultaneously in an aqueous medium, but for less hydrophilic or hydrophobic surfaces, such as unactivated silica (Si–O–Si and Si–OH) or graphene, transferred electrons can make up more than 80% of the surface charge.^62^ The obtained surface charge of graphene, regardless of its source, is, of course, crucial for the colloidal stabilization using PEs, and it appears that BPEI, even though metastable over 3 months, with time, starts to bridge graphene particles and sedimentation occurs. It has been shown that PEI can reduce GO and at the same time be covalently grafted to the reduced GO sheets at 80 °C in a time frame of 2 h.^64^ A similar reaction may occur at a longer storage time at room temperature, which reduces the stabilizing action of BPEI over time.

**Figure 3:**
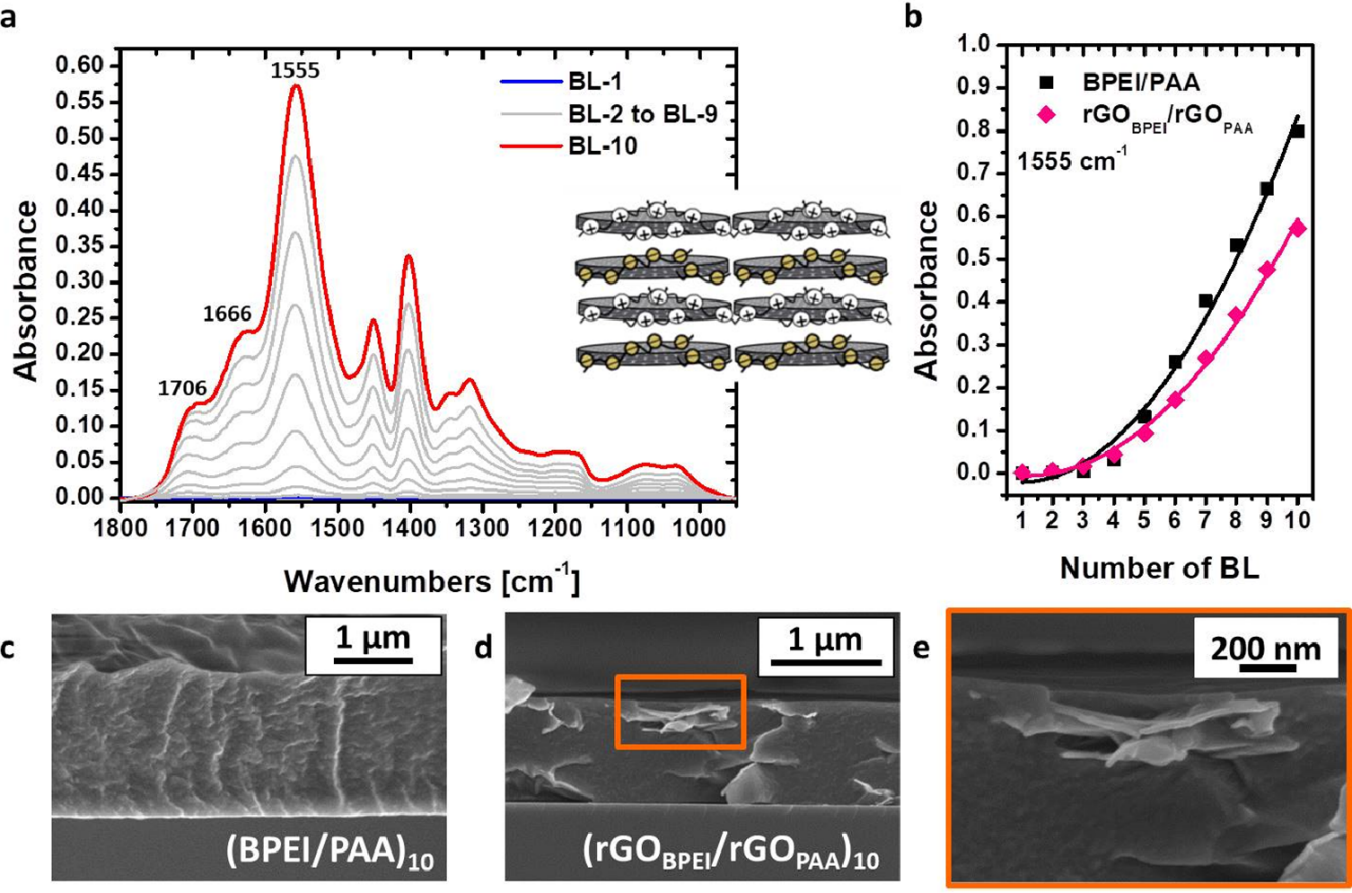
LbL growth of the rGO_BPEI_/rGO_PAA_ system monitored by FT-IR spectroscopy (a). Comparison between BPEI/PAA and rGO_BPEI_/rGO_PAA_ LbL regime growth, based on NH_3_^+^ bending absorption at 1555 cm^–1^ (b). Cross-sectional micrograph of 10-BL BPEI/PAA (c) and 10-BL rGO_BPEI_/rGO_PAA_ (d) assemblies; high-magnification FESEM micrograph of rGO embedded in the rGO_BPEI_/rGO_PAA_ matrix.

The force on separation in Figure 3d provides further insight into the dispersing action of PAA. When the double-layer barrier is overcome, there is a strong interaction between PAA and graphene, although not as strong as in the case of BPEI, which can be seen from the pull-off force. During the dispersing procedure, the energy input from tip sonication is enough for PAA to come into close contact with graphene and form a negatively charged complex. The energy barrier is lower at a lower pH when the carboxylic groups are partially protonated and uncharged.

Figure 2e,f shows the relationship between the pull-off force and electrolyte concentration, and in both cases, 1 mM NaCl seems to be a special condition, that is, the minimum pull-off force for BPEI and maximum pull-off force for PAA. The reason for this is unclear, but it is probably related to the properties of the charged groups or the architecture of the polymers (branched vs linear) since BPEI and PAA show opposite behavior. Another efficient stabilization mechanism for carbon nanoparticles is achieved by charged nanocelluloses.^65^ It has been proposed that the charge of the nanocellulose induces a polarization of the sp^2^ carbon lattice, which leads to an ion–electron correlation force and the formation of stable complexes.^66^ A similar polarization effect could also be considered here although the mechanism is supposed to be more complex as it involves structural factors such as mobility/flexibility of PEs versus rigid cellulose nanoparticles.

### LbL Assembly

The LbL assembly by an alternate deposition of rGO_BPEI_ and rGO_PAA_ dispersions was monitored by FT-IR spectroscopy and QCM-D on Si wafers and quartz crystals, respectively. The characteristic of FT-IR signals for BPEI and PAA (see Figure S5 and Table S2 for peak assignment of BPEI and PAA) can be recognized in the spectra of rGO_BPEI_/rGO_PAA_ BL (Figure 3a). The most intense absorption at 1555 cm^–1^ is ascribed to the deformation of protonated amines in BPEI combined with the symmetric stretching vibration mode of COO^–^ in PAA, along with shoulders at 1706 and 1666 cm^–1^, assigned to C=O stretching and COO^–^ asymmetric stretching, respectively, of carboxylate functionalization in PAA.^67^ The intensity of these signals increases proportionally to the deposited BL number, indicating the occurrence of LbL assembly for rGO_BPEI_/rGO_PAA_, similarly to the BPEI/PAA spectrum evolution with the number of BLs, as reported in Figure S6.

By plotting the absorbance of the signal at 1555 cm^–1^ as a function of BL number, it is apparent that the rGO_BPEI_/rGO_PAA_ system, similarly to the reference BPEI/PAA, follows a superlinear growth regime (Figure 3b). This is in agreement with previously reported literature studies dealing with BPEI/PAA self-assembled coatings.^34,68^ This behavior is attributed to the pH sensitivity of functional groups of weak PEs and the charge overcompensation. The PAA solution used for deposition has a pH of 4 and the ionization degree of the neat PAA is <5% and hence a significant amount of the carboxyl groups of the adsorbed PAA exists in the −COOH form. Subsequently, when the deposited PAA layer is immersed in the BPEI solution, it is exposed to a basic pH of ∼9 that promotes the dissociation of COOH groups to COO^–^, contributing to an increase in the available charge and an increased absorbance in the 1555 cm^–1^ band.^68−70^ Similarly, BPEI experiences an increased charge density when exposed to the acidic PAA solution. Once the charge density of the polymers is increased, more counter-charged polymer groups are needed for the compensation, but since the adsorbing polymer is in a low-charge state due to the pH, indirect overcompensation occurs. This also means that the presence of a weakly charged PE will affect the degree of dissociation/protonation of the other PEs in the layer, as previously described.^70^ As this process continues, with each deposition step, more BPEI and PAA are adsorbed, resulting in a nonlinear growth of film thickness as a function of deposited layers.^71,72^ While both BPEI/PAA and rGO_BPEI_/rGO_PAA_ display a superlinear growth regime, differences in FT-IR signal growth (Figure 3b) suggest that BPEI/PAA assembly grows thicker than rGO_BPEI_/rGO_PAA_ at comparable layer numbers. This can be ascribed to the presence of rGO partially limiting the diffusion and interpenetration of polymer chains through the assembly, as previously reported in the literature for “exponentially” growing LbL encompassing inorganic sheets.^73^

FESEM micrographs of 10-BL cross sections (Figure 3c,d) support the effect of rGO on the LbL growth, based on thickness values of 1.2 ± 0.3 and 1.0 ± 0.1 μm for (BPEI/PAA)_10_ and (rGO_BPEI_/rGO_PAA_)_10_ coatings, respectively. While both assemblies appear continuous, (BPEI/PAA)_10_ yields a more wrinkled inner structure and the (rGO_BPEI_/rGO_PAA_)_10_ cross section shows the presence of rGO highly oriented parallel to the surface, which is evident from high-magnification micrographs (Figure 3e).

**Figure 4:**
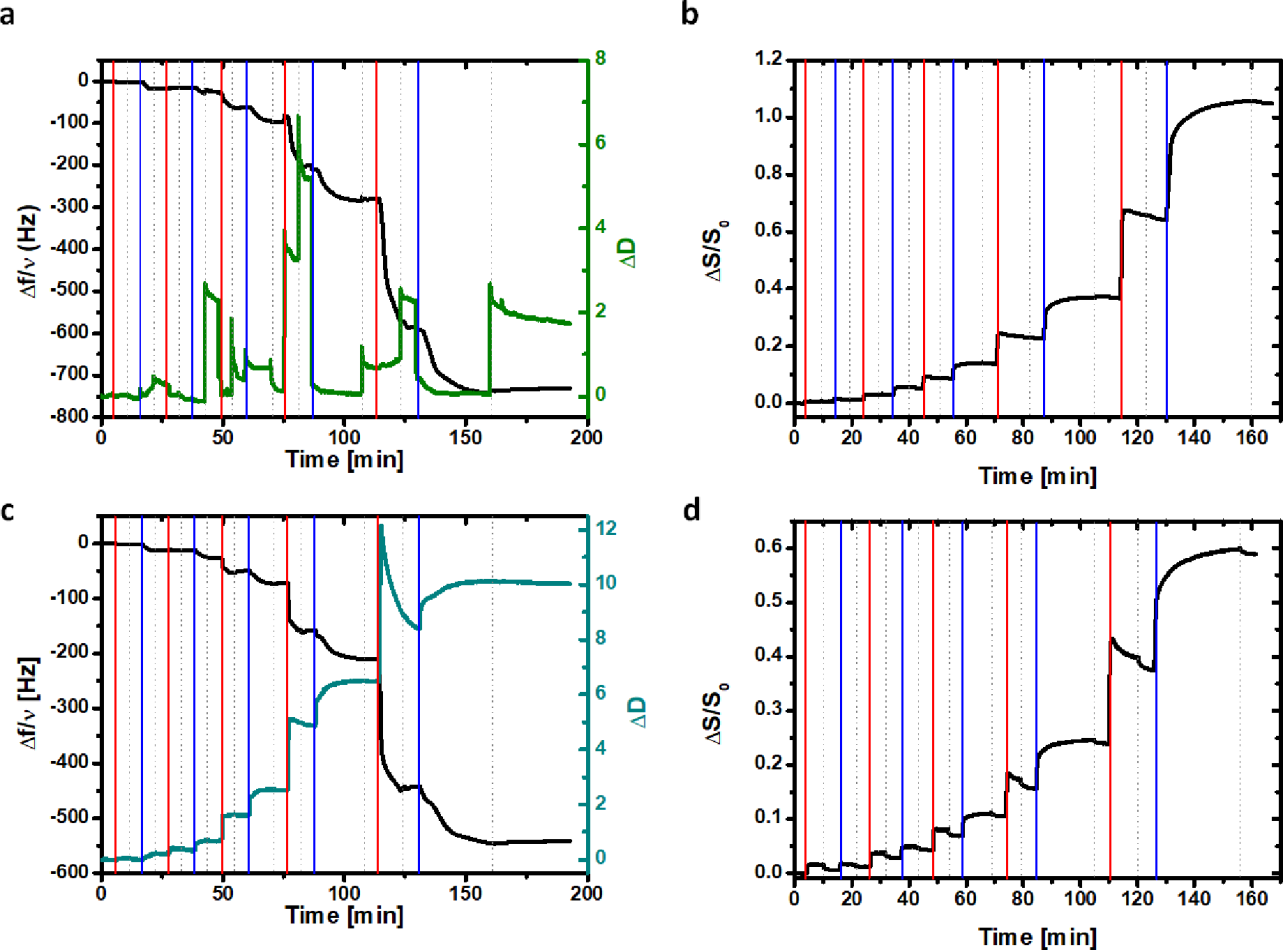
Frequency and dissipation (a) by QCM-D, and SPAR (b) LbL-monitored growth of (BPEI/PAA)_5_ assembly. Frequency and dissipation (c) by QCM-D, and SPAR (d) LbL-monitored growth of (rGO_BPEI_/rGO_PAA_)_5_ assembly. In the graphs, vertical red lines refer to the adsorption of BPEI or rGO_BPEI_ dispersion in BPEI-PAA or rGO_BPEI_/rGO_PAA_ assembly and vertical blue lines refer to the adsorption of PAA or rGO_PAA_ in BPEI/PAA or rGO_BPEI_/rGO_PAA_ assembly. Dotted gray lines refer to washing with ultrapure water.

To further investigate the LbL assembly in real time, QCM-D was used to study depositions of up to 5-BL BPEI/PAA and 5-BL rGO_BPEI_/rGO_PAA_. In both (BPEI/PAA)_5_ and (rGO_BPEI_/rGO_PAA_)_5_ systems, a progressive decrement in the quartz sensor oscillation frequency (Figure 4a–c) was observed over time, which can be ascribed to the increased mass during LbL assembly. The quartz crystal oscillation frequency decreased at each deposition step and the reduction was more pronounced as the number of deposition steps increases, which is a typical behavior of a superlinear regime growth,^74^ corroborating what was already observed in FT-IR experiments for both assemblies.

The dissipation curve (Figure 4a), showing the energy dissipated during quartz crystal sensor oscillation as a function of time during the LbL growth of BPEI/PAA assembly, suggests that the situation is further complicated by the presence of water inside the film. Indeed, every time a new layer of PAA is deposited, the dissipation value falls to almost 0, due to the release of water, which leads to a collapse of the assembly as suggested by the partial decrement in dissipation. The oscillating dissipation probably represents the migration of water in and out of the films due to the change in charge balance in the film as a result of the shift between pH values of 4, 6, and 9 when the different PE solutions or the rinsing medium are introduced.

When rGO is embedded within the coating, the frequency shift (Figure 4c) is lower than the shift for the (BPEI/PAA)_5_ assembly, and the dissipation signals are more continuous than in the BPEI/PAA LbL assembly. However, considering the dissipation (Figure 4c), this is true for the first three BLs because, starting from the fourth BL, the dissipation starts to show some oscillation, which might indicate migration of water. The combination of these observations suggests that (rGO_BPEI_/rGO_PAA_)_5_ assembly is less rigid than the reference, given the more consistent energy loss evidenced at each oscillation, typical of a more viscous layer. To avoid the contribution of water to the measured adsorbed mass during LbL buildup, SPAR was used to study *in situ* the assembly of both (BPEI/PAA)_5_ and (rGO_BPEI_/rGO_PAA_)_5_ coatings. Since SPAR is an optical technique, the reported signal (Δ*S*/*S*_0_) is related to the refractive index increment in the adsorbed material. Because rGO both absorbs and reflects light, the acquired signal is presented as a qualitative comparison. Figure 4b–d shows that the increase in the Δ*S*/*S*_0_ signals at each deposition step is more pronounced as the number of deposition step increases, supporting a superlinear regime growth, as already observed in FT-IR and QCM experiments for both assemblies. The ratio between the SPAR signal and the frequency shift in QCM ((Δ*S*/*S*_0_)/Δ*f*) is 1.4 × 10^–3^ in the case of (BPEI/PAA)_5_ assembly and 1.1 × 10^–3^ in the case of (rGO_BPEI_/rGO_PAA_)_5_ multilayers, which qualitatively shows that the involvement of rGO leads to a gradual accumulation of immobilized water since the adsorbed layer cannot relax due to the rigidity of rGO. This is also indicated by the higher dissipation value in the case of the (rGO_BPEI_/rGO_PAA_)_5_ multilayers.

Differently from QCM-D experiments, the washing step is responsible for a slight decrease in the Δ*S*/*S*_0_ signals that could be ascribed to the removal of weakly bound PEs or rGO. This can be observed in both (BPEI/PAA)_5_ and (rGO_BPEI_/rGO_PAA_)_5_ assemblies, but the latter seems to be more sensitive to this effect.

To complement the AFM colloidal probe study and to determine the adsorption amount for these measurements, we assembled multilayers of the PEs and rGO using SPAR (freshly prepared rGO dispersion, without stabilizers). Figure S7a shows initially high adsorption of BPEI onto the negatively charged silica wafer. The subsequent adsorption of alternating rGO and BPEI layers leads to a moderate but steady increase in adsorbed mass, which agrees with the “electrostatic” interaction observed in the colloidal probe measurements. In contrast, the LbL assembly of PAA and rGO, after the initial BL of polyallylamine hydrochloride (PAH)/PAA, shows that there is hardly any increase in adsorbed mass due to the repulsion between the negatively charged rGO and PAA (Figure S7b). Since the adsorption is only governed by diffusion toward the surface, there is not enough energy to overcome the double-layer barrier and achieve van der Waals attraction. The slight increase is probably due to just a rearrangement of the PAH/PAA layer when PAA is reintroduced or a minor baseline drift.

### Gas Barrier Properties

Both (BPEI/PAA)*_n_* and (rGO_BPEI_/rGO_PAA_)*_n_* coatings were deposited on a 10 μm PET substrate to improve its gas barrier properties. SEM observations (Figure S8a,b) do support the thickness trend observed on model silica and quartz surfaces, with (BPEI/PAA)_5_ resulting in a thicker coating than the (rGO_BPEI_/rGO_PAA_)_5_. The latter has a slightly rougher surface morphology, likely due to the presence of rGO embedded within the PE matrix.

**Figure 5:**
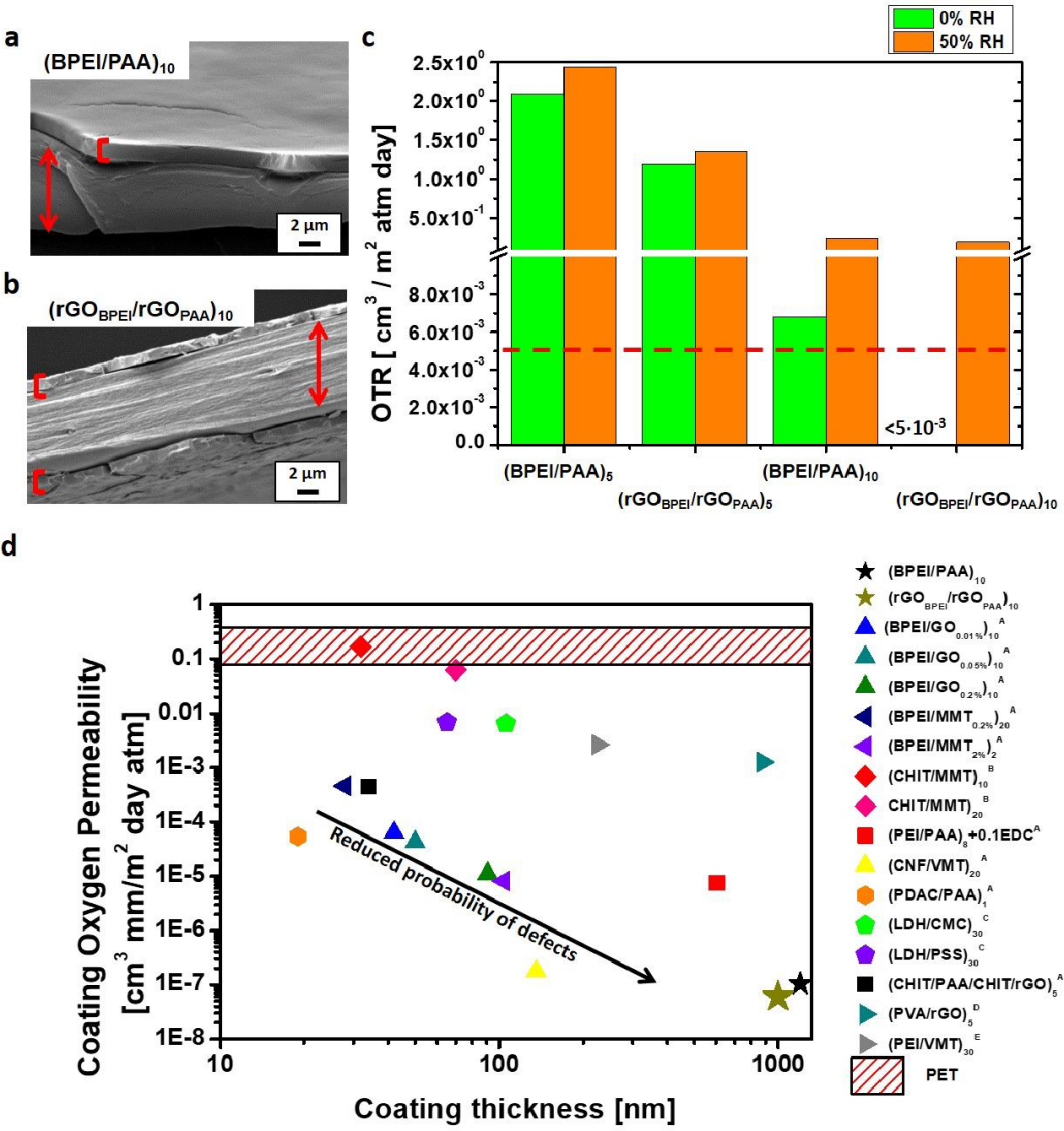
SEM micrograph of a PET, 10 μm thick, coated by (BPEI/PAA)_10_ (a) and (rGO_BPEI_/rGO_PAA_)_10_ (b) assembly. PET is labeled in red arrow and coating thickness is highlighted with red square brackets. The cracks showed in SEM micrographs are due to the fracture of the sample conducted in liquid nitrogen. OTR measurements collected on (BPEI/PAA)_5_, (BPEI/PAA)_10_, (rGO_BPEI_/rGO_PAA_)_5_, and (rGO_BPEI_/rGO_PAA_)_10_ samples (c), and dotted line represents the sensitivity limit of the instrument. Comparison between LbL coatings developed in the literature and (BPEI/PAA)_10_ and (rGO_BPEI_/rGO_PAA_)_10_ systems (d). Comparison between LbL coatings developed in the literature and (BPEI/PAA)_10_ and (rGO_BPEI_/rGO_PAA_)_10_ systems (d). ^A^Performed on PET 179 μm, with a permeability of P 1.518 [cm^3^ mm/(m^2^ day atm)] RH 0%; 0.01%, 0.05%, 0.2%, and 2% indicate the weight percent concentration of the lamellar filler used for LbL assembly, ^B^coatings deposited on PLA 500 μm thick, ^C^coatings deposited on PP 120 μm thick, ^D^coatings deposited on PET 23 μm thick, ^E^coating deposited on biaxial-oriented PP 17.8 μm thick, ()*_n_* indicates the number of deposited BLs. (BPEI/GO_wt%_)*_n_*,^33^ (BPEI/MMT_wt%_)*_n_*,^75^ (CHIT/MMT_wt%_)*_n_*,^35^ (PEI/PAA)_8_ + 0.10EDC,^76^ (CNF/VMT)_20_,^77^ (PDAC/PAA)_complex_,^78^ (LDH/CMC)_30_ and (LDH/PSS)_30_,^37^ (CHIT/PAA/CHIT/rGO)_5_,^79^ (PVA/rGO)_5_,^45^ and (PEI/VMT)_30_;^80^ permeability data were calculated from the literature. The dashed area in (d) indicates the range of neat PET permeability, as reported in ref (81).

Pristine PET exhibits OTR values of 134 and 121 cm^3^/(m^2^ day atm) at 0% and 50% RH, respectively. After the LbL deposition of both BPEI/PAA or rGO_BPEI_/rGO_PAA_ coatings, the gas barrier properties toward oxygen improve drastically, with 3–4 orders of magnitude reduction in OTR (Figure 5c). Furthermore, under dry conditions, rGO_BPEI_/rGO_PAA_-coated PET films exhibit significantly better performances than the corresponding BPEI/PAA at the same BL number. Interestingly, the (rGO_BPEI_/rGO_PAA_)_10_ PET film exhibits an OTR below 0.005 cm^3^/(m^2^ day atm), which corresponds to the sensitivity limit of the instrument. Under humid conditions, (rGO_BPEI_/rGO_PAA_)_5_ coating still performs better than (BPEI/PAA)_5_, whereas at 10 BL, an equivalent performance was obtained for both coatings. This can be related to the moisture sensitivity of the multilayers. Indeed, under humid conditions, the coatings tend to swell because of the hydrophilic nature of the polymers. Both BPEI and PAA can trap water, introducing free volume accessible to oxygen and consequently lowering the barrier efficiency.^76^ The presence of rGO limits the interpenetration of the polymer backbones and also reduces the possibility of trapping water, thus contributing to maintaining an efficient physical barrier to oxygen diffusion until 5-BL deposition.^35,75^ By increasing the number of deposited layers, the amount of PEs in each of the layers is higher than that for 5 BLs, as a consequence of the superlinear regime growth, and under these conditions, the presence of rGO appears to be insufficient to significantly limit the absorbance of water and the loss in barrier properties.

By considering the parameters affecting permeability and the recent literature background,^45^ it seems that the decrease in the diffusivity coefficient produced by highly oriented and impermeable rGO is the main factor responsible for the observed increase in barrier properties compared to the BPEI/PAA assembly.

The performances of the obtained samples were compared to others in the literature containing BPEI or chitosan (CHIT), as positive PEs, and GO or montmorillonite (MMT), as negatively charged nanoplates. To properly compare performances for coatings with different thicknesses and on different substrates, the permeability values of the coatings, decoupled from the overall permeability of the substrate with the coating, were calculated according to earlier studies^82^ and are shown in Figure 5d and Table S3. The inclusion of rGO in the multilayers improves the barrier efficiency of the rGO_BPEI_/rGO_PAA_-deposited coatings, yielding a permeability 5 times lower than that of BPEI/PAA coatings under 50% RH conditions (Table S3). Comparing this result with other LbL assemblies (Figure 5d and Table S3), it is clear that the permeability of coatings prepared in this work is well below the previously reported permeability of 10–20 BL coatings of other PE/nanoplate assemblies. Indeed, the (rGO_BPEI_/rGO_PAA_)_10_ coating permeability turned out to be 2–3 orders of magnitude lower than the best systems based on MMT or GO. This behavior can be ascribed to the obtained “brick-and-mortar” structure, which results in a new stratification configuration where nanoparticles are strictly connected to the polymer backbone and included within the assembly at every deposition step. Different from the previous literature, where nanoplates are deposited alternatively to a polymer layer, in this paper, we presented the deposition of rGO tightly coupled with both BPEI and PAA at every deposition step resulting in a doubled deposition rate of nanoplates. Moreover, the presence of polymers on both sides of the rGO allows the deposition of a layer consisting of sandwiched rGO with PE complexes in between, leading to a brick-and-mortar-fashioned coating with a high polymer content (see inset in Figure 3a). The advantage is not only that a higher mass can be adsorbed in each deposition step but also that the complexed polymer “mortar” phase probably results in films with fewer defects, as suggested by the outstanding gas barrier performance. Figure 5d also shows that the coating permeability decreases with increasing thickness of the coating, which is probably due to a reduced probability of defects in the coating.

Permeability to water vapor was also evaluated on 10-BL-treated samples, as this represents a complementary set of information to oxygen permeability required for many applications. Water vapor transmission rates (WTRs) (measured at 23 °C/50 RH and 38 °C/90 RH) and the calculated permeability values are reported in Table S4. The presence of the coating is responsible for a reduction in water vapor permeability. The best results are achieved by rGO-containing assemblies with 32% and 15% reductions compared to neat PET at 23 °C/50 RH and 38 °C/90 RH, respectively. By contrast, the 10-BL BPEI/PAA coating yields limited performances (i.e. 19% and 6% at 23 °C/50 RH and 38 °C/90 RH, respectively). These results further highlight the improved barrier performances achieved, thanks to the presence of rGO. A comparison with other high oxygen and water vapor barrier technologies points out that the developed (rGO_BPEI_/rGO_PAA_)_10_ coating is capable of competing with some of the best packaging solutions currently applied in practice such as EVOH films, metalized polymer laminates, and SiO*_x_* coatings (Table S5).^83^

## Conclusions

This paper presents a viable route for the stabilization of reduced GNPs in water-based solutions using BPEI and PAA as positively and negatively charged PEs, respectively. These polymers yielded dispersions stable up to 3 months and 1 year, respectively. The dispersions were further used for the fabrication of LbL self-assembled coatings. To investigate the mechanism of interactions, experiments with a graphene model surface were carried out by AFM colloidal probe measurements in combination with SPAR data of rGO and PE assemblies and show that graphene obtains a negative charge in water, which leads to attraction with BPEI and repulsion with PAA. However, in the case of PAA, when the double-layer barrier is overcome by energy from tip sonication, van der Waals interactions allow stabilization regardless of the like-charge configuration. The rGO_PE_ dispersions enable LbL assembly with superlinear growth, yielding thick coatings where rGO was preferentially oriented parallel to the substrate surface and embedded within the two PE assemblies. Coatings consisting of 10 BLs improved the gas barrier properties of thin PET films obtaining an OTR value below 5 × 10^–3^ cm^3^/(m^2^ day atm), in 0% RH. In addition, the presence of rGO limited the interpenetration of polymers and lowered the swelling of the coating in 50% RH. These coatings could deliver significantly better barrier performances than previous examples in the literature for similar assemblies, comprising layered silicate or GO nanoplates. The PE-assisted stabilization of nanoplates opens future possibilities for rGO water-based nanocomposite assemblies that would otherwise be impossible. The high mass deposited in each step also enables rapid buildup, which is a major challenge for LbL assembly at scale.

## Experimental Section

### Materials

rGO was obtained via oxidation of graphite, ultrasonication, and the subsequent thermal reduction of GO. A water dispersion of GO was prepared using a modified Hummers’ method in H_2_SO_4_. Starting from large flakes of natural graphite (provided by NGS-Naturgraphit, Leinburg, Germany) and using a proportion of graphite/KMnO_4_/NaNO_3_ of 1:4:0.25, the reaction temperature was kept between 0 and 6 °C for 24 h. Following this, the resulting solution was slowly heated to 20 °C and maintained at this temperature for 72 h of reaction. To remove the excess of MnO_4_^–^, an H_2_O_2_ solution was added to the reaction mixture and stirred overnight. After sedimentation, the solution was washed with a 4 wt % HCl solution under mechanical stirring for 2 h. The solid was filtered, and the obtained wet GO was dispersed in water and stirred at 1000 rpm for 30 min. This dispersion was tip-sonicated with a UP400S Hielscher (Potsdam, Germany) for 60 min using a sonotrode H22 with 100% of amplitude and full-cycle condition. The GO was then purified by centrifugation at 4000 rpm and then thermally reduced in an oven in an argon atmosphere at 1060 °C obtaining the rGO (from now labeled as pristine rGO; *I*(D)/*I*(G): 1.30 ± 0.07; C/O content: 49; SSA: 196 m^2^/g). Dispersion and solutions were prepared using ultrapure water having a resistance of 18.2 MΩ, supplied by a Q20 Millipore system (Milano, Italy). PAA (solution average *M*_w_ ∼ 100,000 g/mol, 35 wt % in H_2_O, CAS: 9003-01-4) and BPEI (*M*_w_ ∼ 25,000 g/mol by laser scattering, *M*_n_ ∼ 10,000 g/mol by gel permeation chromatography, as reported in the material datasheet, CAS: 9002-98-6) were purchased from Merck (Milano, Italy). Used oxygen and nitrogen gases (pureness 5.5) were purchased from Rivoira (Turin, Italy). PET films (amorphous, 10 μm thick) were used as substrates for the preparation of LbL gas barrier films. PET films were cleaned with deionized water to remove deposited dust and ethanol to remove excess water, and the films were subsequently dried in an oven at the temperature of 70 °C for 2 min.

### Preparation of rGO Dispersions

Pristine rGO (25 mg) was added to 100 mL of PAA (pH 4.5) or BPEI (pH 8.5) solution (0.1 wt %) and ultrasonicated at 150 W for 15 min, applying an impulse of 30 s on/30 s off (i.e., resulting in a total of 15 min on and 15 off) (Sonics, Vibra-cell-VCX-50, 13 mm tip, Newtown, USA). This process was repeated after 5 min of cooling. The obtained colloidal dispersion was centrifuged at 3300 rpm for 30 min (Eppendorf, Centrifuge 5702, Hamburg, Germany) and left for decantation overnight. The supernatant was collected the next day, in the form of a black dispersion, from now on denoted as rGO_PAA_. This procedure was applied to 100 mL of PE solution/25 mg of rGO batches until 1 L of dispersion was obtained. The same procedure was applied for the preparation of GNP_BPEI_ colloidal dispersion, from now on denoted as rGO_BPEI_.

### LbL Deposition

Single-side-polished (100) silicon wafers were used as a model substrate for monitoring the LbL growth by FT-IR spectroscopy. The silicon wafer was alternately dipped in the rGO_BPEI_ (pH 8.5) and rGO_PAA_ (pH 4.5). After each deposition step, the substrate was washed with ultrapure water jet and dried under room-temperature compressed airflow. The first BL was achieved with a dipping time of 10 min, while the time was reduced to 1 min for each of the BL up to 10. The same procedure was applied for the deposition of the BPEI/PAA assembly.

PET films (10 μm thick) were alternatively dipped in the rGO_BPEI_ and rGO_PAA_, washed with water jet, and dried in a ventilated oven at 70 °C after each deposition step. The dipping time was set to 10 min for the first BL deposition and decreased to 1 min for the following ones. The process was repeated until 5 and 10 BLs were deposited, and the obtained samples were labeled as (rGO_BPEI_/rGO_PAA_)_5_ and (rGO_BPEI_/rGO_PAA_)_10_, respectively.

### Characterization

SEM experiments were carried out using an LEO-1450VP SEM (imaging beam voltage: 5 kV, Jena, Germany). AFM was performed on an Innova AFM by Bruker (Bremen, Germany), equipped with RTESPA-300 tapping mode probes with a resonant frequency of 200–400 kHz and a spring constant of 20–80 N/m. The colloidal dispersions of rGO_BPEI_ and rGO_PAA_ were diluted at 1:10 in ultrapure water and deposited dropwise on a standard silicon double-sided 285 nm SiO_2_ wafer. A reference dispersion of rGO was obtained by the tip sonication of 25 mg of the pristine rGO in ultrapure water, following the procedure described above. Topography maps by AFM were obtained by depositing one layer of rGO by dipping the silicon substrate in a water dispersion for 5 min and drying it in air. Raman spectra were obtained on an InVia Raman microscope (Renishaw, New Mills, UK; argon laser source 514 nm/50 mW, 10 scansions) coupled with a Leica DM 2500 optical microscope. The reported spectra of rGO, either pristine or PE functionalized, are normalized to the D band at 1354 cm^–1^. The rGO concentration in rGO_BPEI_ and rGO_PAA_ dispersions was determined by TGA (Q500 by TA Instruments, Newcastle, USA; weight sensitivity ±0.1 μg, dynamic baseline drift ±50 μg calculated by the producer using empty platinum pans in the range of temperature 50–1000 °C with 20 °C/min, no baseline correction, and a temperature sensitivity of ±0.01 °C). An aliquot of both dispersions was dried in an oven, and about 8 mg was employed to perform the measurements and analyzed in the range of 100–800 °C at 10 °C/min in a N_2_ atmosphere. The rGO concentration was calculated assuming that the concentration of the employed PE remains constant through the stabilization process, and there is no interaction between the components during decomposition (i.e., each component decomposes independently). With these hypotheses, the concentration of the solution was evaluated by performing a calculated TG (i.e., a weighted average of the TG of the neat components) to match the experimental residue measured for the dried dispersion, thus obtaining the amount of rGO responsible for the increase in the final residue.

For the AFM colloidal probe measurements, silica wafers covered by a single layer of graphene were purchased from Graphenea (San Sebastián, Spain), delivered in square pieces of 10 × 10 mm^2^, and were used as received. Tipless cantilevers with a spring constant on the order of 0.3 N/m were purchased from MikroMasch (Wetzlar, Germany) and were calibrated in air under ambient conditions using the AFM tune IT 2.5 software (Force IT, Sweden). Silica particles with a radius of 5 μm (Duke Standards, dry borosilicate glass microspheres, Thermo Scientific) were glued to cantilevers using an earlier reported protocol.^84^ The dimensions of the particles were measured using an optical microscope. BPEI and PAA were adsorbed for 10 min *in situ* in the AFM liquid cell at a concentration of 0.1 g/L at pH 7 using a silica wafer as support and with the colloidal probe present. The probe was rinsed after and in between the adsorption steps using 0.01 mM NaCl (TraceSELECT, Sigma-Aldrich). The silica wafer was then replaced by the graphene model surface, and the colloidal probe was dried with an air gun to consolidate the adsorbed PE layer before rewetting and force measurements. Force curves were recorded in 0.01, 0.1, 1, and 10 mM NaCl in a 5 × 5 array with 500 nm between each measurement location. Typical force curves are presented, and pull-off forces are presented as the minimum, mean, and maximum values due to quite large variations at different locations on the graphene surface. The LbL assemblies were monitored by FT-IR spectroscopy (PerkinElmer Frontier, Waltam, USA; 32 scans, 4 cm^–1^ resolution, transmission mode) using the single-side polished (100) Si wafer as the substrate. Cross sections of LbL-coated Si wafers were imaged by high-resolution FESEM (Zeiss Merlin 4248, Jena, Germany; beam voltage: 5 kV). Samples were chromium sputtered before FESEM observations.

QCM-D (E4 model, Q-Sense AB, Gothenburg, Sweden) was used to study the LbL growth. The sensor crystals (silicon oxide, QSX 303 SiO_2_, Q-Sense AB, Gothenburg, Sweden) were cleaned with Milli-Q water and EtOH before activation in the oxygen plasma (PDC 002, Harrick Scientific Corp., Ossining, NY, USA). QCM experiments were carried out at the constant temperature of 24 °C with a flow rate of 0.15 mL/min and with a concentration of 0.1 g/L of BPEI (pH 7.5), PAA (pH 4.5), rGO_BPEI_ (pH 7.4), and rGO_PAA_ (pH 4.3). The fifth harmonics have been reported for both normalized frequency variations (Δ*f*/ν) and dissipation (Δ*D*).

SPAR was used to measure the adsorbed amount in each layer of rGO_BPEI_ or rGO_PAA_ during LbL assembly. Since SPAR is an optical technique, water is not included and the signal represents dry mass in contrast to QCM, in which the mass of strongly bound water is included. The SPAR experiment was carried out at a constant temperature of 23 °C with a concentration of 0.025 g/L of BPEI (pH 7.0), PAA (pH 4.6), rGO_BPEI_ (pH 7.0), and rGO_PAA_ (pH 4.6). Milli-Q water was used for rinsing, and all solutions and dispersions were used at native pH. Adsorption time and rinsing time were set to 5 min or until a plateau was reached. The details of the technique have been described elsewhere.^85^ The presented signal Δ*S*/*S*_0_ is proportional to the refractive index change at the interface, and the adsorbed mass is related to the refractive index increment of the adsorbed material (d*n*/d*c*). rGO both reflects and absorbs light, which makes it challenging to determine the actual adsorbed mass without using too many assumptions in the Fresnel equation. The obtained data was thus used for qualitative comparisons only.

The cross sections of LbL-treated PET films were studied by SEM imaging, as already described in this section. LbL-coated PET films were immersed in liquid nitrogen, cracked into two pieces, one of which was pinned up on conductive adhesive tapes and gold-sputtered before SEM imaging. The gas barrier properties of untreated and LbL-treated films were evaluated by permeability measurements on a 100 cm^2^ film surface and were measured using a MOCON OX-TRAN 2/21 Module SH (Neuwied, Germany). The OTR was evaluated in 0% and 50% of RH conditions at 23 °C. The experimental relative error was estimated to be within ±1%. The WTR of untreated and LbL-treated films was evaluated on aluminum foil masked samples (exposed area, 1 cm^2^) using a MultiPerm apparatus (Extra Solutions, Lucca, Italy). The experimental relative error was estimated to be within ±10%. The coating contribution to the permeability of the LbL-treated PET films was calculated, as reported in the literature, from the permeability of the composites (PET + coating) applying the equation:
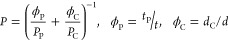
where *t*_P_, ϕ_P_, and *P*_P_ are the thickness, volume fraction, and permeability of the PET, respectively, while *t*_C_, ϕ_C_, and *P*_C_ are the thickness, volume fraction, and permeability values of the coating layers, respectively.^82^

## Supporting Information Available

SEM images of pristine rGO; TGA in the nitrogen atmosphere of neat BPEI, PAA, and pristine rGO; TGA residues of neat polymer and suspensions; SEM and AFM of rGO_BPEI_ and rGO_PAA_; AFM colloidal probe data between a silica probe and a single-layer graphene; FT-IR signal attribution for PAA and BPEI; FT-IR spectra of PAA and BPEI; FT-IR LbL growth of (BPEI/PAA)_10_ assembly; SPAR data of the growth of 3-BL PE-rGO assembly; comparison between LbL coatings developed in the literature and BPEI/PAA and rGO_BPEI_/rGO_PAA_ systems; SEM micrograph of a PET, 10 μm thick, coated by (BPEI/PAA)_5_ and (GNP_BPEI_/GNP_PAA_)_5_ assembly; water vapor transmission rates and water vapor permeability values performed at 23°C/50% RH and 38°C/90% RH of uncoated and coated PET films; and oxygen and water vapor permeability for other barrier technologies (PDF)
