# Supplementary material for: Polyelectrolyte-Assisted Dispersions of Reduced Graphite Oxide Nanoplates in Water and Their Gas-Barrier Application
Source: ACS Appl Mater Interfaces. 2021 Sep 3;13(36):43301–13. doi: 10.1021/acsami.1c08889 (PMC8447182; doi:10.1021/acsami.1c08889)
Supplement: Supplementary file 1 — am1c08889_si_001.pdf [file am1c08889_si_001.pdf]

Supporting information

Polyelectrolyte-Assisted Dispersions of Reduced Graphite Oxide Nanoplates in Water and Their Gas-Barrier Application

Lorenza Maddalena [a], Tobias Benselfelt [b], Julio Gomez [c], Mahiar Max Hamedi [b], Alberto Fina [a], Lars Wagberg [b]*, Federico Carosio [a]*

[a] Dipartimento di Scienza Applicata e Tecnologia, Politecnico di Torino, Alessandria Campus, Viale Teresa Michel 5, 15121 Alessandria, Italy

[b] Department of Fibre and Polymer Technology, KTH Royal Institute of Technology, Teknikringen 58 SE-100 44 Stockholm, Sweden

[c] AVANZARE Innovacion Tecnologica S.L., 26370 Navarrete, La Rioja, Spain

*Corresponding authors:

e-mail: wagberg@kth.se, Tel: +46 8 790 82 94;

e-mail: federico.carosio@polito.it, Tel: +39 0131 22930.

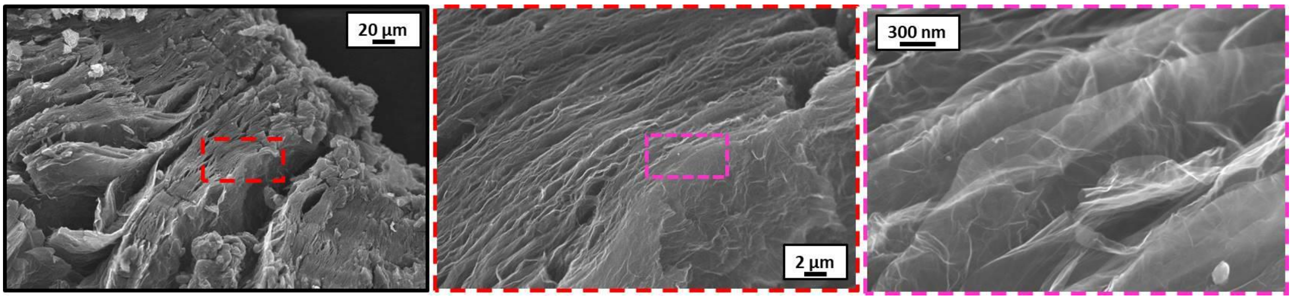

Figure S 1 SEM images of pristine rGO

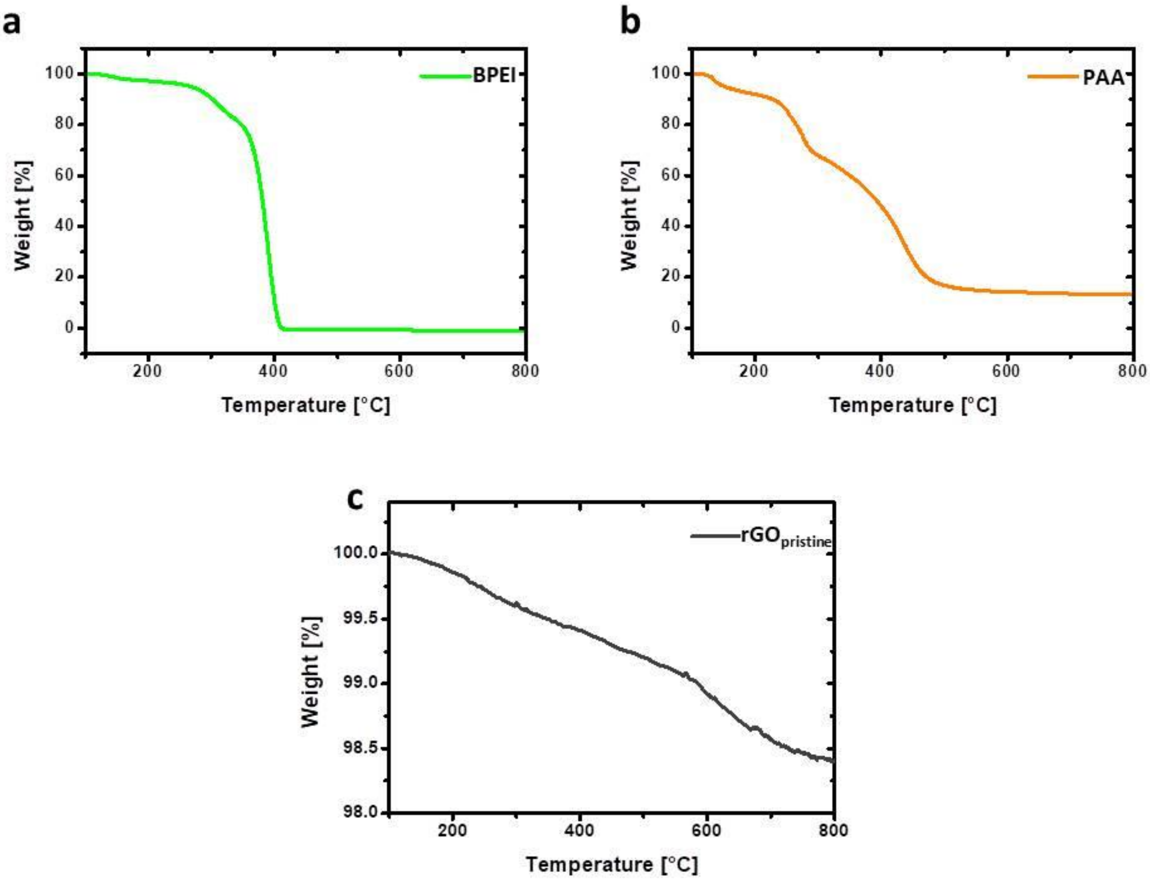

Figure S 2 TGA in nitrogen atmosphere of neat BPEI, PAA and pristine rGO.

Table S 1 TGA residues of neat polymer and suspensions

Sample	Residue [%]	Sample	Residue
**rGO pristine**	98.4		
**BPEI**	0	GNP_{BPEI}	3.9
**PAA**	13.1	GNP_{PAA}	14.8

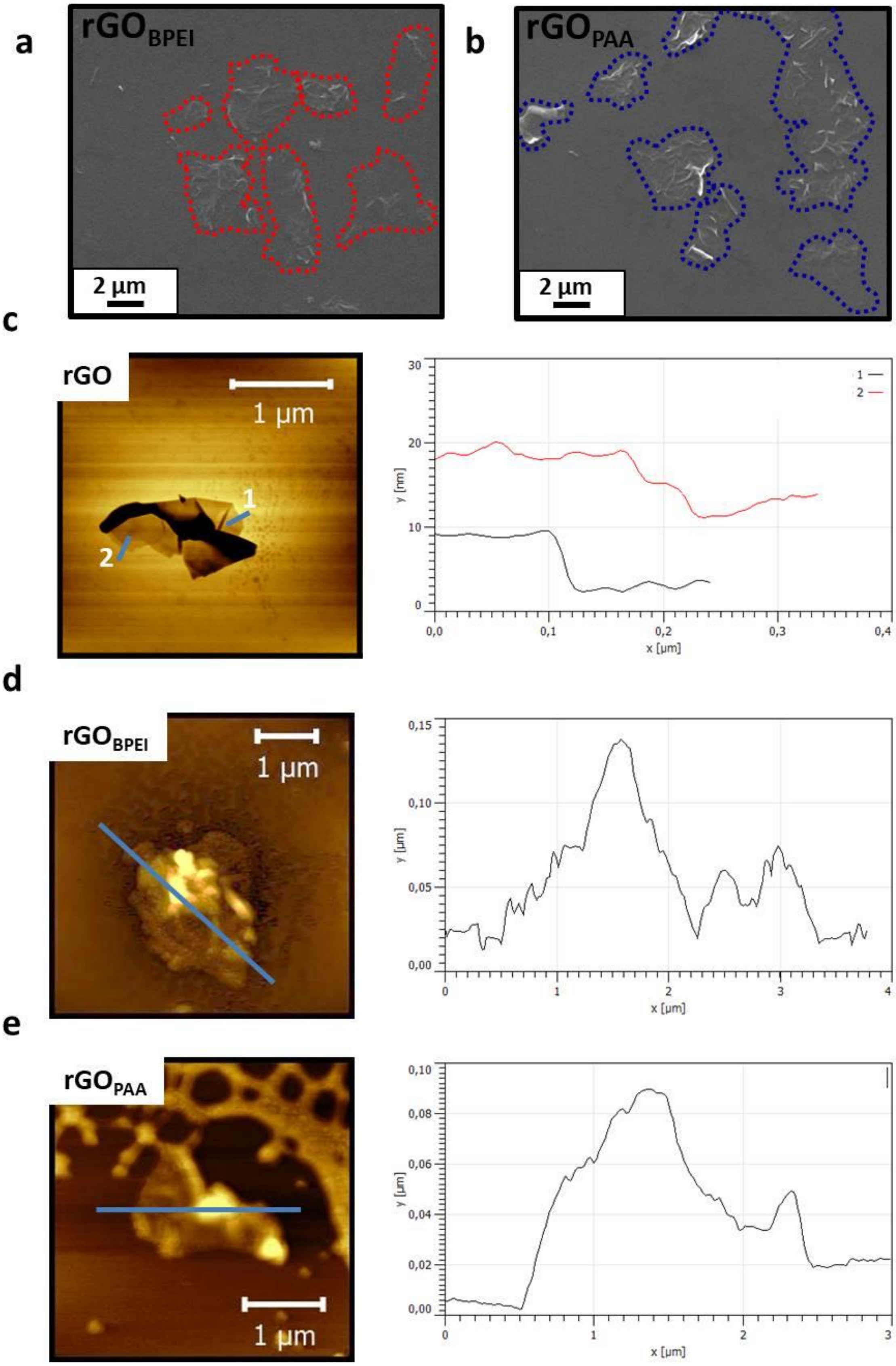

Figure S 3 SEM micrographs of rGO_{BPEI} (a) and rGO_{PAA} (b) dried suspensions deposited on a Si wafer. Note that in (b) some rGO appears to be overlapped each other. AFM tapping mode topography of a single rGO (c), a single rGO covered by BPEI in dried rGO_{BPEI} suspension (d), a single rGO covered by PAA in dried rGO_{PAA} suspension.

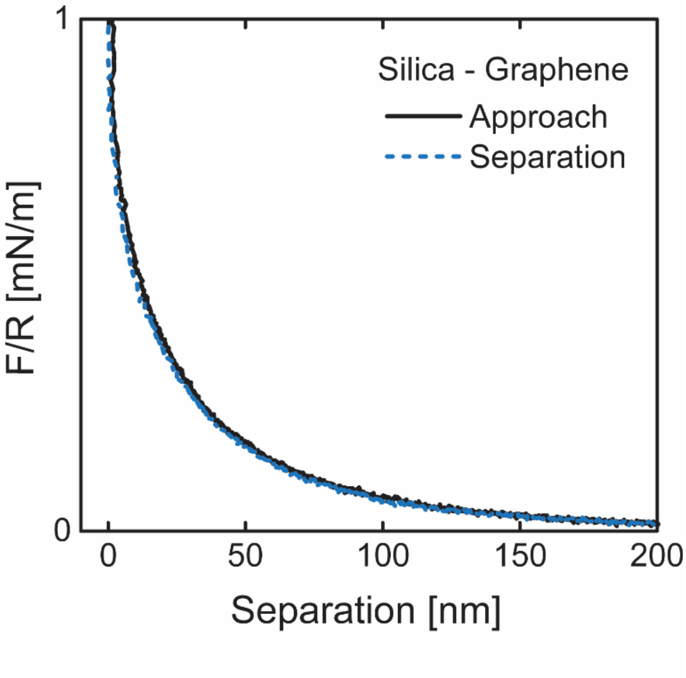

Figure S 4 AFM colloidal probe data of the interaction between a silica probe and a single-layer graphene surface on approach and separation. The electrolyte concentration was 0.01 mM NaCl.

Table S 2 FT-IR signal attribution for PAA and BPEI

PAA			BPEI		
**Absorbance [cm^{-1}]**	**Signal**	**Attribution**	**Absorbance [cm^{-1}]**	**Signal**	**Attribution**
**3455**	A	OHν_{as} of water	3368	J	NH ν_{as}
**3180**	B	OHν_{as} of PAA	3299	K	NH ν_s
**2954**	C	$CH_2\nu_{as}$ and CHν_{as}	2959	L	$CH_2\nu_{as}$ and CHν_{as}
**2880 and 2591**	D	Overtones and combination of bands H and I enhanced by Fermi resonance with the broad OH ν_{as} band	2849	M	$CH_2\nu_s$ and CHν_s
**1714**	E	C=Oν_s	1570	N	NH δ_I
**1453**	F	CH_2 deformation	1477	O	NH δ_{II}
**1415, 1255 and 1180**	G	C-Oν_s coupled O-H $\delta_{in\ plane}$	1413	P	CH_3 ν_s of secondary and tertiary amines
**1111**	H	C-CH_2 ν	1312	Q	CH_2 $\delta_{wagging}$
**804**	I	C-CH_2 δ_{twist} and C-COOH ν	1281	R	CH_2 $\delta_{twist-rocking}$
			1154	S	CN ν_{II}
			1108 and 1011	T	CH_3 $\delta_{rocking}$
			1049	U	CN ν_I
			817	V	NH_2 $\delta_{wagging}$

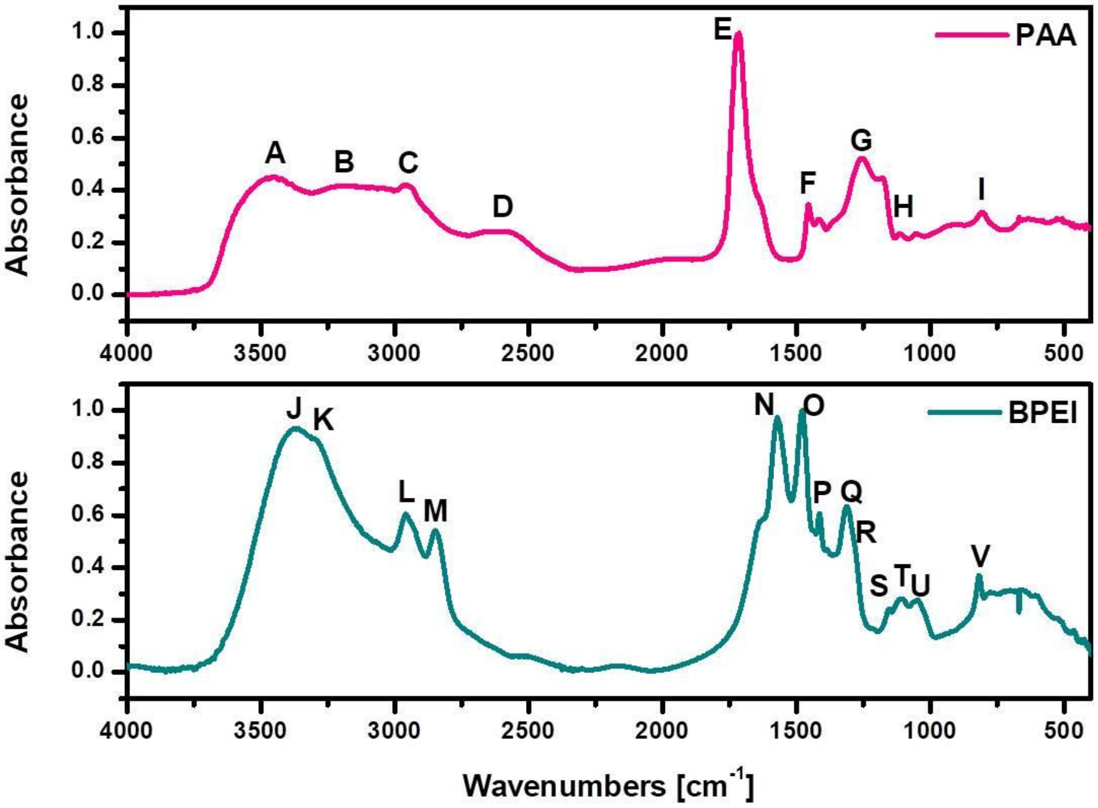

Figure S 5 FT-IR spectra of PAA and BPEI

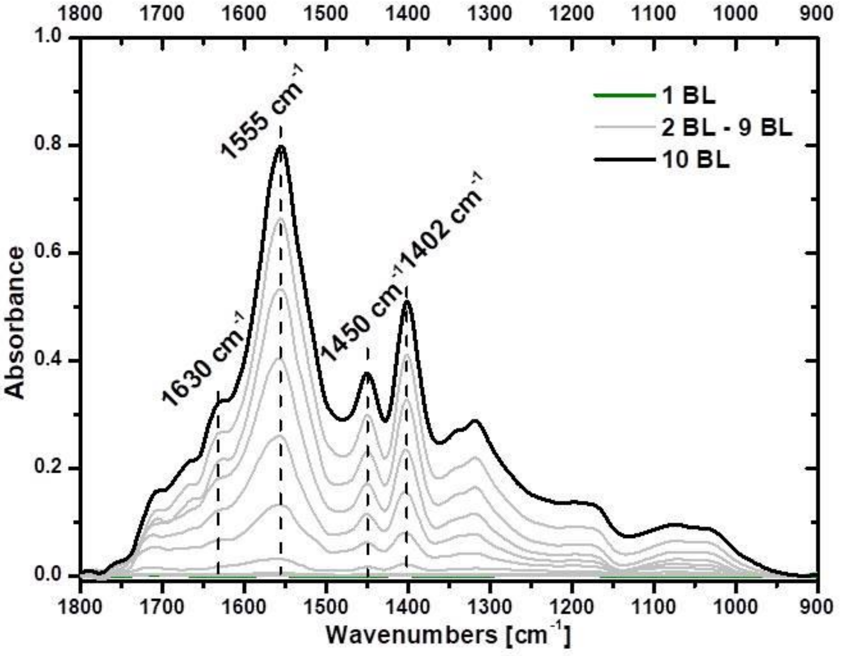

Figure S 6 FTIR LbL growth of (BPEI/PAA)$_{10}$ assembly

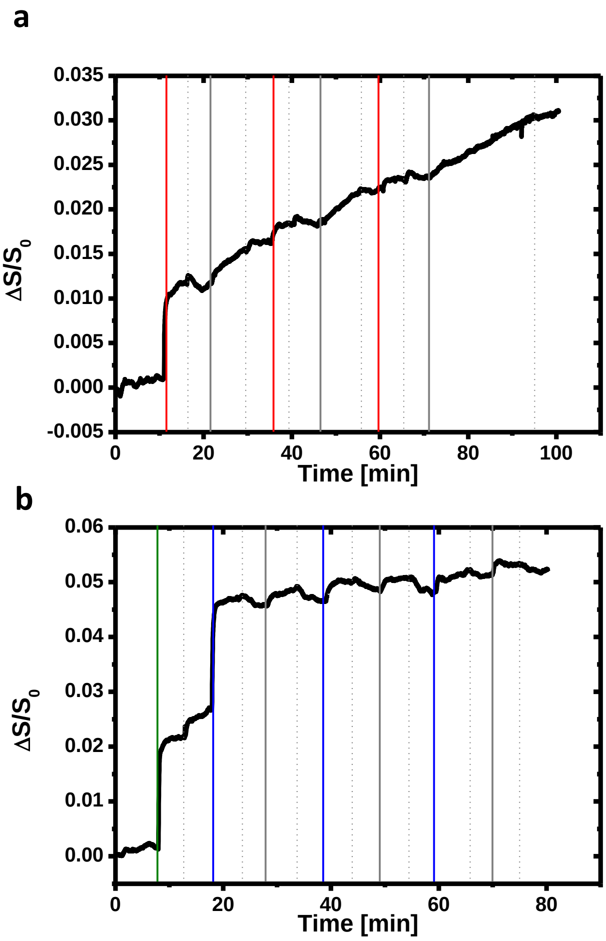

Figure S 7 SPAR data of the growth of 3 BL PE-rGO assembly showing (a) $(rGO/BPEI)_3$ LbL and (b) $(rGO/PAA)_3$ LbL. In the graphs vertical red lines means the adsorption of BPEI in BPEI/rGO assembly and vertical blue lines means the adsorption of PAA in rGO/PAA assembly. Grey vertical lines indicate rGO adsorption while dotted grey lines means washing with ultrapure water. The green line in (b) represent an anchoring layer of polyallylamine hydrochloride (PAH).

Table S 3 Comparison between LbL coatings developed in the literature and BPEI/PAA and rGO_{BPEI} /rGO_{PAA} systems

Sample	Coatings thickness [nm]	OVERALL Oxygen Permeability [cm³ mm/(m² day atm)] RH 0%	COATING Oxygen Permeability [cm³ mm/(m² day atm)] RH 0%	OVERALL Oxygen Permeability [cm³ mm/(m² day atm)] RH 50%	COATING Oxygen Permeability [cm³ mm/(m² day atm)] RH 50%	Ref.
**PET 10 µm**	-	1.34		1.21		This
**$(BPEI/PAA)_{10}$**	1200	8.7 x 10^{-5}	1.042 x10^{-7}	0.003	1.8*10^{-3}	work
**$(rGO_{BPEI}/rGO_{PAA})_{10}$**	1000	< 6.00 x 10^{-5}	< 6.000 x 10^{-8}	0.002	3.7*10^{-4}	
**$(BPEI/GO_{0.01\%})_{10}^{*}$**	42	0.229	6.332 x 10^{-5}	nd	nd	[33]
**$(BPEI/GO_{0.05\%})_{10}^{*}$**	50	0.138	4.235 x 10^{-5}			
**$(BPEI/GO_{0.2\%})_{10}^{*}$**	91	0.022	1.108 x 10^{-5}			
**$(BPEI/MMT_{0.2\%})_{20}^{*}$**	28	1.033	4.618 x 10^{-4}	nd	nd	[75]
**$(BPEI/MMT_{2\%})_{20}^{*}$**	104	0.014	8.179 x 10^{-6}			
**$(CHIT/MMT)_{10}$**	32	1.414	1.696 x 10^{-1}	nd	nd	[35]
**$(CHIT/MMT)_{20}$**	70	0.281	6.232 x 10^{-2}			
**$(PVA/rGO)_5$**	882	0.175	1.251 x 10^{-2}	nd	nd	[45]
**$(PEI/VMT)_{30}$**	226	1.044	2.652 x 10^{-2}	nd	nd	[80]
**$(PEI/PAA)_8$+0.10 EDC**	603	0.001	7.483 x 10^{-6}	nd	nd	[76]

**(CNF/VMT)$_{20}$**	136	23.3 x 10^{-4}	1.770 x 10^{-7}	nd	nd	[77]
**(PDAC/PAA)$_{complex}$**	19	0.005	5.395 x 10^{-5}			[78]
**(LDH/CMC)$_{30}$**	106	0.019	6.486 x 10^{-3}	nd	nd	[37]
**(LDH/PSS)$_{30}$**	65	0.020	6.882 x 10^{-3}			
**(CHIT/PAA/CHIT/rGO)$_5$**	34	0.061	4.452 x 10^{-4}	nd	nd	[79]

[*] 0.01%, 0.05%, 0.2% and 2% indicate the %wt concentration on lamellar filler used for LbL assembly, ()$_n$ indicates the number of deposited bi-layers. (BPEI/GO%wt)$_n$,[33] (BPEI/MMT%wt)$_n$,[75] (CHIT/MMT%wt)$_n$,[35] (PEI/PAA)$_8$ +0.10EDC,[76] (CNF/VMT)$_{20}$,[77] (PDAC/PAA)$_{complex}$,[78] (LDH,/CMC)$_{30}$ and (LDH/PSS)$_{30}$,[37] (CHIT/PAA/CHIT/rGO)$_5$,[79] (PVA/rGO)$_5$,[45] (PEI/VMT)$_{30}$, [80] permeability data were calculated from literature. The dashed area in (d) indicate the range of neat PET permeability as reported in ref 81.

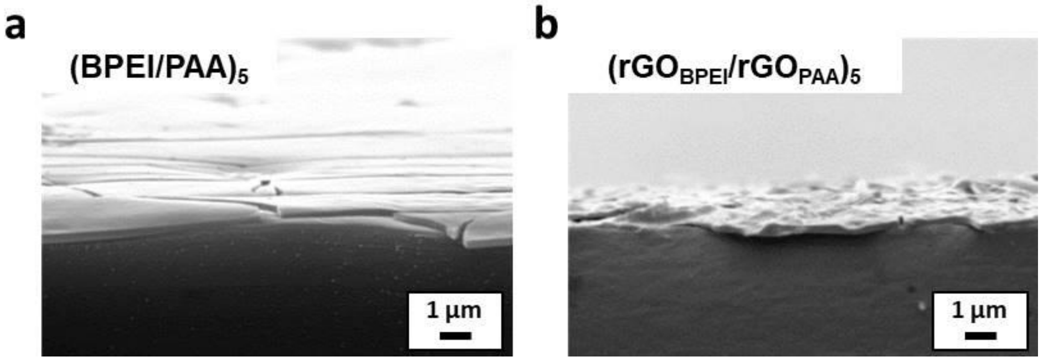

Figure S 8 SEM micrograph of a PET 10 µm thick coated by (BPEI/PAA)$_5$ (a) and (GNP$_{BPEI}$ /GNP$_{PAA}$)$_5$ (b) assembly.

Table S 4 Water Vapor Transmission Tate (WVTR) and Water Vapor Permeability of PET, (BPEI/PAA)$_{10}$ and (rGO$_{BPEI}$/rGO$_{PAA}$)$_{10}$ assembly at 50%RH-23°C and 90%RH-38°C.

Sample	WVTR [g/m^2 day], 50%RH, 23°C	P [g mm/m^2 day atm], 50%RH, 23°C	WVTR [g/m^2 day], 90%RH, 38°C	P [g mm/m^2 day atm], 90%RH, 38°C
**PET 10 µm**	1.6 x 10^1	0.16	6.0 x 10^1	0.60
**(BPEI/PAA)$_{10}$**	1.0 x 10^1	0.13	4.6 x 10^1	0.56
**(rGO$_{BPEI}$/rGO$_{PAA}$)$_{10}$**	0.90 x 10^1	0.11	0.40 x 10^1	0.51

Table S 5 Oxygen and water vapor permeability for other barrier technologies

	Oxygen Permeability in cc·mm /(m^2 day atm)	Water vapor permeability in g·mm/ '(m^2 day atm)	Calculated from reference
**EVOH[a]**	0.001–0.01	40–120	
**Polymer laminate 67 μm (PET/EVOH/PE)[b]**	0.1	7-20	
**Metallised laminate 62 μm (PET/Met/PE)[b]**	0.06-0.12	0.7-2.1	**83**
**SiO$_x$ coating on 12 μm PET[b]**	0.006-0.06	0.15-4.3	

[a] 23°C 0% relative humidity (R.H.) for oxygen permeability and at 23°C 85% R.H. for water vapor permeability;
[b] 23°C 50% R.H. for both oxygen and water vapor permeability;
